# Subaqueous geomorphology and delta dynamics of Lake Brienz (Switzerland): implications for the sediment budget in the alpine realm

**DOI:** 10.1186/s00015-021-00399-1

**Published:** 2021-12-20

**Authors:** Stefano C. Fabbri, Isabel Haas, Katrina Kremer, Danae Motta, Stéphanie Girardclos, Flavio S. Anselmetti

**Affiliations:** 1 Institute of Geological Sciences, Oeschger Centre of Climate Change Research, University of Bern, Baltzerstr. 1+3, 3012 Bern, Switzerland; 2 Swiss Seismological Service, ETH Zurich, Sonneggstrasse 5, 8092 Zurich, Switzerland; 3 Department of Earth Sciences/Institut Des Sciences de L’Environnement (ISE), University of Geneva, Rue des Maraîchers 13, 1205 Geneva, Switzerland

**Keywords:** Alpine lake, Sediment budget, Quasi-4D reflection seismic data, Subaquatic delta channel, Megaturbidites

## Abstract

Non-invasive techniques such as seismic investigations and high-resolution multibeam sonars immensely improved our understanding of the geomorphology and sediment regimes in both the lacustrine and the marine domain. However, only few studies provide quantifications of basin wide-sediment budgets in lakes. Here, we use the combination of high-resolution bathymetric mapping and seismic reflection data to quantify the sediment budget in an alpine lake. The new bathymetric data of Lake Brienz reveal three distinct geomorphological areas: slopes with intercalated terraces, a flat basin plain, and delta areas with subaquatic channel systems. Quasi-4D seismic reflection data allow sediment budgeting of the lake with a total sediment input of 5.54 × 10^6^ t sediment over 15 years of which three-quarter were deposited in the basin plain. Lake Brienz yields extraordinarily high sedimentation rates of 3.0 cm/yr in the basin plain, much more than in other Swiss lakes. This can be explained by (i) its role as first sedimentary sink in a high-alpine catchment, and by (ii) its morphology with subaquatic channel-complexes allowing an efficient sediment transfer from proximal to distal areas of the lake.

## Introduction

(Peri-)alpine lakes play a major role as sedimentary sinks and represent important archives for studying past environmental and climatic conditions (Glur et al., 2015; Jenny et al., 2014; Leemann & Niessen, 1994; Lister et al., 1983; Rapuc et al., 2019; Wirth et al., 2011), sediment transport (e.g. Chapron et al., 2002; Arnaud et al., 2016; Silva et al., 2019), and seismotectonic activity (Kremer et al., 2017; Moernaut et al., 2018; Rapuc et al., 2018; Van Daele et al., 2019). Understanding the geomorphology of (peri-)alpine lake basins helps to unravel sedimentation patterns and thus provides insights into Quaternary landscape evolution and glaciation history of the Alps (Fabbri et al., 2018; Glur et al., 2015; Hilbe et al., 2011). In oligotrophic lakes, sedimentation is mostly dominated by allochthonous clastic input of the major tributaries, which in turn can be linked with denudation processes in the catchment (Anselmetti, Bühler, et al., 2007; Sturm & Matter, 1978). A crucial role in sedimentation processes can be attributed to the delta areas acting as transition zone between the subaerial and the subaquatic domain (Silva et al., 2019). In systems with high riverine sediment discharge, slope failures in deltas and turbidity currents occur often leading to erosion of the substrate (Girardclos et al., 2012; Hizzett et al., 2018) and formation of subaquatic-channel complexes (Corella et al., 2014). Those features, being a small-scale analogue of submarine canyons along continental margins, can be considered as effective conduit transferring high amount of sediments from proximal to distal areas (Corella et al., 2016; Kremer, Corella, et al., 2015). Understanding this highly dynamic environment that sometimes produces catastrophic delta failures (Hilbe et al., 2011), can further help to improve natural hazard assessment and implementing safety measures for lakeshores as well as coastal communities.

In this study, a combined approach of newly acquired high-resolution bathymetric and reflection seismic data provides profound insights into geomorphology and sediment dynamics of Alpine Lake Brienz. Furthermore, a quasi-4D comparison of seismic data from this study with older vintages from 2003 (Girardclos et al., 2007) allows budgeting of basinal sediment accumulation in the past 15 years. We eventually compare the sediment budget calculated from quasi-4D seismic reflection data with instrumental dataset of the annual sediment load of major rivers.

## Geologic setting and previous studies

**Fig. 1:**
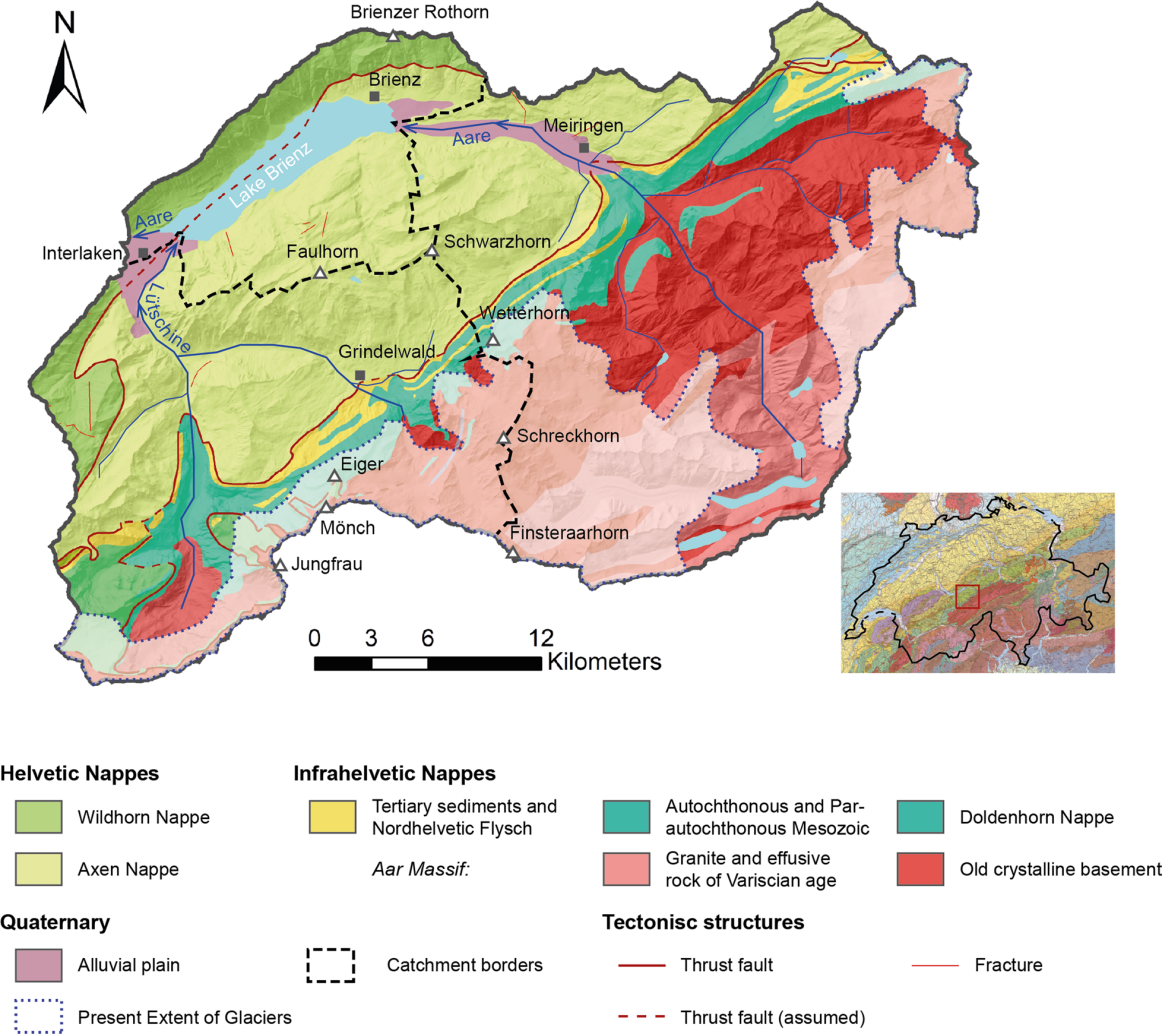
Tectonic map (superimposed on shaded relief using swisstopo swissALTI3D digital elevation model) of Lake Brienz’ catchment area. Sub-catchment borders of Lütschine and Aare are represented by black dashed lines. Inset shows tectonic map of Switzerland (GK500-Tekto, swisstopo)

Lake Brienz is an alpine lake at the frontal range of the Bernese Swiss Alps. The lake is formed in a local overdeepening carved out by the Aare Glacier over several glacial cycles (Preusser et al., 2010). Due to its high-altitude alpine catchment and low population density, Lake Brienz is poor in nutrient input and can be considered as ultraoligotrophic (BAFU, 2016; Sturm & Matter, 1978). Lake Brienz sediments are characterized by a δ^13^C value of calcite that is ca. 1‰, pointing to a detrital origin of lake carbonates from the Mesozoic limestones (Bechtel & Schubert, 2009). Thus, sedimentation in the lake is almost exclusively dominated by allochthonous clastic input of its main tributaries Aare and Lütschine, forming large delta areas on either end of the lake (Adams et al., 2001). Fine-grained sediment yield, originating from glacial meltwater in spring and summer, turns the lake colour into a milky turquoise (Jordi et al., 2006, p. 4). Lateral steep mountain torrents on the other hand play a minor role in the sedimentary budget of the lake (Sturm & Matter, 1978). The fjord-like lake covers an area of 29.8 km^2^ and has a catchment area of ~ 1100 km^2^ (Table 1). The catchment of the lake is in an Alpine-tectonic context entirely situated within the Helvetic domain and the Central Massifs belonging to the former passive margin of the European continent (Pfiffner, 2011). Lake Brienz is surrounded by two nappe complexes: the Wildhorn nappe comprising Cretaceous to Cenozoic sedimentary rocks on the northwestern shore, and the Axen nappe composed of Jurassic sedimentary rocks on the southeastern shore (Rowen, 1993; Fig. 1). A major thrust fault, striking parallel to the longitudinal axis of the basin, separates the two tectonic units (Hänni & Pfiffner, 2001). In the higher catchment area, crystalline rocks of the Aar Massif are separated from the Helvetic nappes by a narrow band of Tertiary sedimentary rocks belonging to the Infrahelvetic complex (Hänni & Pfiffner, 2001). The catchment lithology is also represented in the mineralogic composition of the lake’s main tributaries. The Lütschine River, which drains mostly sedimentary terrain, brings high input of carbonates to the lake. Sediments from the Aare River, on the other hand, with large areas of the catchment covering crystalline bedrock of the Aar Massif, are mostly of siliciclastic origin (Sturm, 1976).

**Table 1:** Physical properties of Lake Brienz and its catchment (BAFU, 2016, 2018)

Altitude of lake level	564 m a.s.l	Catchment area	1138 km^2^
Surface area	29.8 km^2^	Mean altitude of catchment	1951 m a.s.l
Volume	5.15 km^3^	Average water discharge Aare (2018)	37.1 m^3^/s
Maximum length	14.2 km		
Maximum width	2.8 km	Average water discharge Lütschine (2018)	19.0 m^3^/s
Mean depth	173 m		
Maximum depth	256 m		
Mean residence time water	2.6 yrs

Several seismic surveys of Lake Brienz have been completed in the past years. In 1969 and 1971, Matter et al. (1973) acquired 30 km of airgun and 70 km of high-resolution boomer and mud-penetrator data (see Matter et al., 1973 and Leenhardt, 1964 for more details on the method). These seismic data revealed two troughs containing 300 and 550 m thick sediment packages. Maximum bedrock depth was located at 800 m below lake level, indicating that glacial erosion reached ~ 230 m below modern sea level (Matter et al., 1973). In 1998 and 2003, high-resolution seismic data were acquired using a 3.5 kHz pinger source (Schmidt, 1998; Adams et al., 2001; Girardclos et al., 2007). The hitherto most extensive seismic survey was carried out by Girardclos et al. (2007): A 350-m spaced grid of pinger seismic lines totaling a length of 240 km covering the entire Lake Brienz area was used as a basis for seismic stratigraphic analysis of the basin fill.

First bathymetric measurements of Lake Brienz were done by the Swiss Federal Office of Topography in the years 1866 and 1898. Later in 1932/33 and 1956/57, in connection with the redirecting of the Aare Delta as well as the construction of hydropower dams, more detailed bathymetric studies were done examining morphology of the Aare Delta by the Swiss Federal Bureau of Water Management (Sturm, 1976). The basin morphology with the delta areas and the channel-levee structure was already known at that time and was later described in detail by Matter et al., (1973) and Sturm and Matter (1978).

Sedimentological studies of the lake included several coring campaigns (e.g. Bodmer et al., 1973; Sturm, 1976; Anselmetti, Bühler, et al., 2007; Girardclos et al., 2007). Sturm (1976) studied surface sediments in detail and concluded that Lake Brienz sediment distribution is mainly controlled by the varying stratification of river inflows (over-, inter- and underflows). Sturm (1976) showed that the predominant sediment input is delivered by the two main tributaries, Aare and Lütschine. Based on these findings, a sedimentological model for lacustrine clastic systems has been proposed by Sturm and Matter (1978). In the same studies, mass-movement deposits from both delta areas were described. The authors showed that varve-like sedimentation in the flat basin plain is interrupted by turbidite deposits of several decimeters thickness originating either from the Aare or the Lütschine Deltas. In more recent years, Girardclos et al. (2007) reconstructed a spontaneous collapse that occurred in 1996 at the front of the Aare Delta caused by fast sediment accumulation and overload. The mass-movement deposit extends over an area of ∼8.5 km^2^ with a total volume of 2.72 × 10^6^ m^3^ and amounts to ∼9 yrs of the lake’s entire sediment accumulation (Girardclos et al., 2007). Anselmetti, Bühler, et al. (2007) examined the human influence on the lake’s sedimentation by showing that sediment discharge of the river Aare has been highly altered due to the use of hydropower further upstream: Sediment fluxes to Lake Brienz were reduced by two thirds since sediment is trapped in reservoirs upstream of the Aare. Mostly the coarse-grained deltaic sedimentation is affected. Hemipelagic sedimentation on the other hand remains unaffected. However, a seasonal shift in runoff timing can be observed with increased and decreased particle input in winter and summer, respectively (Anselmetti, Bühler, et al., 2007; Finger et al., 2007; Jaun et al., 2007).

Only few studies provide exact quantification of sediment turnover of subaquatic-channel systems in the lacustrine domain. In earlier studies, sedimentation rates were mostly determined from sediment cores by means of varve counting often combined with radioisotope dating (e.g. Anselmetti, Bühler, et al., 2007; Sturm & Matter, 1972; Von Gunten et al., 1987). Nevertheless, as these are point measurements in three dimensional complex basins, no accurate sediment budgets were obtained. Other studies estimated sedimentation rates from seismic data (Anselmetti, Hodell, et al., 2007; Bini et al., 2007) or comparison of bathymetric data sets (Silva et al., 2019). However, these methods can only provide rough sediment budgets over longer time spans or specific events like mass-movement deposit (e.g. Girardclos et al., 2007) but fall short to resolve sedimentation patterns on decadal timescale.

## Methods

### Bathymetric data

Swath bathymetric data was acquired during a 2-weeks survey in May 2018 using a Kongsberg EM2040 multibeam echo sounder (Kongsberg Maritime, Horten, Norway) operating at a frequency of 300 kHz and a beam width of 1°-by-1°. The motion and orientation of the vessel was recorded with a Seatex MRU5 + motion sensor (Kongsberg Seatex, Trondheim, Norway) and a Trimble SPS361 heading sensor (Trimble Navigation Limited Sunnyvale, CA, USA). A Leica GX1230 GNSS receiver (Leica Geosystems, Heerbrugg, Switzerland) in combination with the swiposGIS/GEO real-time positioning service (Swiss Federal Office of Topography, Wabern, Switzerland) ensured a positioning accuracy of 2–3 cm. A vertical sound-velocity profile with a sound-velocity sensor (Valeport Limited, Totnes, UK) is needed for accurate calibration of the refraction angles in the water column. Sound-velocity changes close to the water surface were permanently monitored using a Valeport MiniSVS sound-velocity sensor (Valeport Limited, Totnes, UK). Data were acquired up to a minimum water depth of 5 m mostly along shore parallel survey track lines in direction of the lake’s longitudinal axis. Recording swath stripes were overlapped to reduce errors in the bathymetric dataset.

Processing of the bathymetric data was performed with CARIS HIPS/SIPS software 10.4 (Caris, Fredericton, Canada). Data corrections were done for navigation and positioning. Recorded artefacts (erroneous lake-bottom detections) were removed manually from the point cloud. Lake-level measurements of the station Brienzersee – Ringgenberg (station number: LH2023) of the Swiss basic hydrological monitoring network were used to correct for lake-level fluctuations during the survey and the dataset was normalized to a long-term (75-years) average lake level of 563.8 m a.s.l. (BAFU, 2018). The resulting digital terrain model has a cell size of 2 m and a vertical resolution of a few centimeters in shallow-water areas and a few decimeters in the deep basin. Additionally, a backscatter-intensity map (cell size 5 m) was computed. The products were interpreted with ArcGIS software.

### Reflection seismic profiles

Seismic data were acquired in October 2018. For quasi-4D analysis, 2D seismic profiles, collected in 2003 (Girardclos et al., 2007), were reproduced. This comprises a longitudinal profile on a SW-NE transect and 10 cross-sections on NW–SE transects with a total length of 20.4 km. The reflection seismic system consists of a 3.5 kHz single-channel pinger source/receiver (GeoAcoustics 3.5 kHz 4-element pinger) built up by four piezoelectric transducer elements. The detailed parameters applied in this survey are summarized in Table 2. The device was fixed on an inflatable catamaran and pushed in front of a motorboat. The digital recording of the seismic data was carried out by an Octopus Marine 760 Shallow Seismic Processor in SEG-Y format. A Garmin GPSmap76Cx GPS receiver (max. error ± 5 m) and Fugawi navigation software (Northport Systems Inc., Toronto, Canada) guaranteed accurate positioning. Seismic data were processed with a bandpass filter (1.7–1.9–6.5–6.9 kHz). Additionally, a bulk shift was applied to the 2018 data to match the vintage of 2003 using a clearly identifiable reference horizon that can be correlated across both vintages. Seismic interpretation was performed with the IHS Kingdom Suite Software 2015 (IHS Inc., Englewood, CO, USA). Two-way travel time (TWT) was converted into depth using an average sound velocity of 1450 m/s confirmed by known depths from the bathymetric dataset. Picked lake bottoms of both seismic datasets from 2003 and 2018 were exported to ArcGIS for calculation of the volume of sediment accumulation. An interpolation of sedimentation rate was done for the area with good 2D seismic line coverage. For data interpretation, only the flat lake basin was considered.

**Table 2:** Applied settings for the high-resolution seismic survey in 2018

Pinger central frequency	3500 Hz
Trigger interval	500 ms
Band-pass filtering	Low cut: 1700 Hz Low pass: 1900 Hz High pass: 6500 Hz High cut: 6900 Hz
Boat velocity	7–8 km/h
Recorded time	500 ms
Sound velocity (water; ~ 10° C; assumed)	1450 m/s
Vertical resolution	¼ wavelength (~ 5–15 cm; resolution decreases with depth)
Max. penetration of the signal	35–40 m below lake floor

## Results

### Bathymetric dataset

#### General lake morphology

**Fig. 2:**
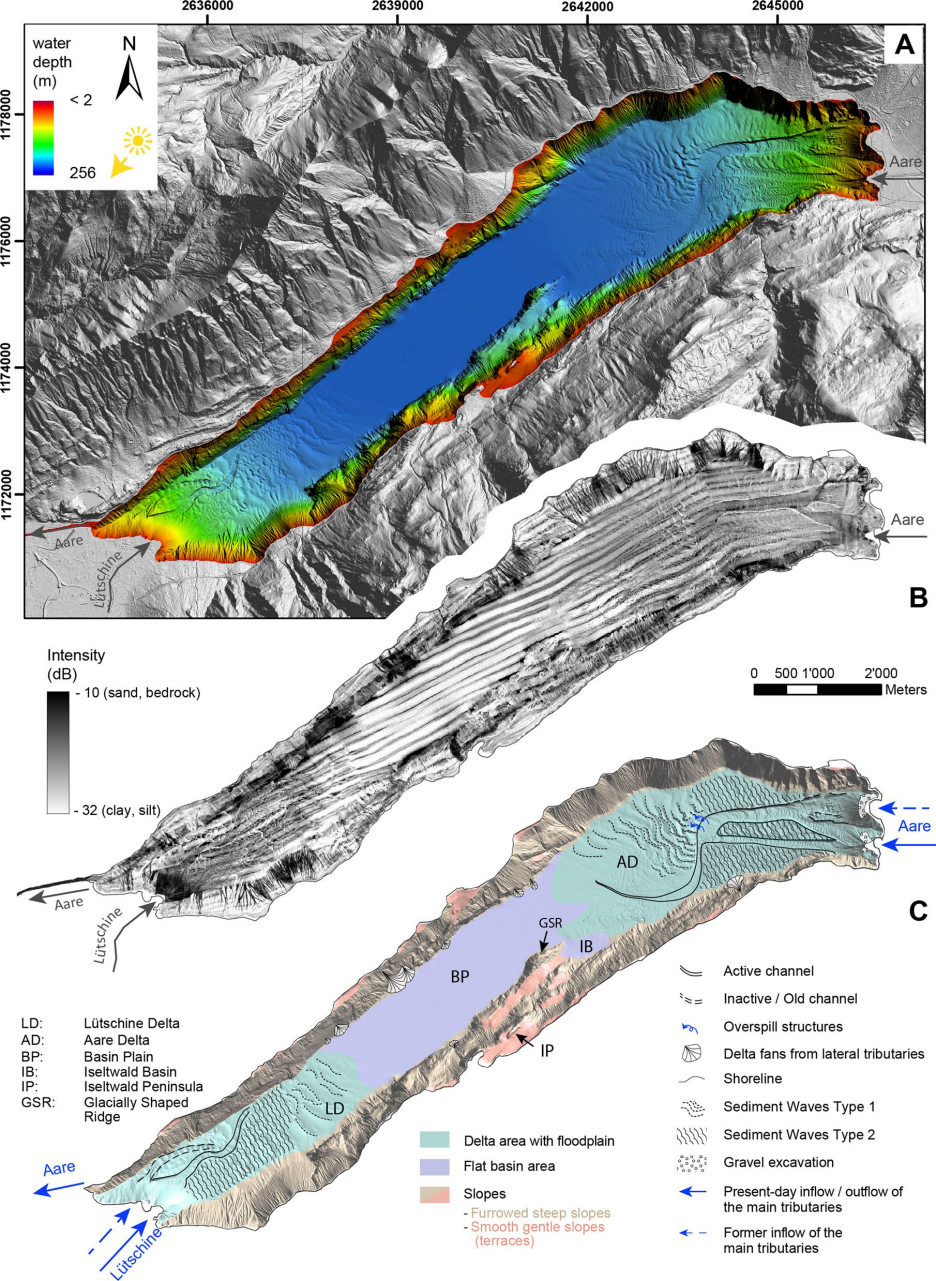
**A** High-resolution bathymetric map of Lake Brienz (2 m grid size, shaded relief with colours representing depth) with shaded relief (swisstopo swissALTI3D digital elevation model) of the catchment. **B** Backscatter-intensity map revealing areas with coarse and hard material (high intensities). Note the basin-parallel data artefacts (dark stripes) which are swath bathymetry related. **C** Interpreted morphology of Lake Brienz

High-resolution bathymetric data reveal a large flat basin area bordered by steep lateral flanks along the north-western and southeastern shoreline, respectively (Fig. 2A). The new bathymetry data prove that the two deltas on either end of the lakes are characterized by subaquatic channels, as described in previous studies (e.g. Sturm & Matter, 1978; Girardclos et al., 2007). The lake can be subdivided into three main geomorphological areas, (i) basin plain, (ii) slopes and (iii) delta areas (Fig. 2C). The basin plain covers 17% of the lake’s total area and reaches a maximum water depth of ~ 256 m (with respect to the long-term average lake level). Its flat topography lacks any current-induced features, in contrast to the delta areas. Further to the northeast, the much smaller Iseltwald Basin can be located (Fig. 2C). The submerged glacially shaped ridge, rising to 90 m above the flat basin plain, and the Iseltwald peninsula, separate the Iseltwald Basin from the rest of the basin plain. The transition from the basin plain to the lateral slopes is sharp and no traces of mass movements can be observed, unlike in many other (peri-)alpine lakes (e.g. Bini et al., 2007; Hilbe et al., 2011; Ledoux et al., 2010). The slopes make up the largest area of the lake (44%). Most of the slopes typically show rough, deeply furrowed topography with steep slope gradients of > 25°. However, a few terraces, intercalated into these steep slopes, are smoother in texture and show gentler slope gradients. A series of steps and terraces can be observed e.g. close to the Iseltwald peninsula (Fig. 2C). These are most probably inherited features from underlying bedrock and/or due to glaciation, as has also been shown for Lake Thun close to Spiez (Fabbri et al., 2018). The shore zone only comprises a narrow band. Locally, the lake is flanked by steep cliffs, particularly along the southeastern shore. Various small delta fans formed by lateral mountain torrents at the slope-basin transition occur.

The remainder of the lake is dominated by the two major delta areas of Aare (25% of lake’s total area) and Lütschine (14% of lake’s total area). The undulated topography is distinct from the flat basin plain and shows prominent features as channels (see Sect. 4.1.2) and current-induced structures like dunes or sediment waves (Fig. 2). Following the classification scheme of Stow et al. (2009), the typical transverse bedforms can be distinguished by their size: dunes (max. 1 m height) are the small-scale analogue of sandwaves (~ 5–10 m height). Sandwaves occur at flow velocities in the range of 0.3 to 0.75 ms^−1^, while sinuous-crested and barchanoid dunes appear in higher flow-velocity regimes of 0.4–0.7 and 0.6–1.2 ms^−1^, respectively (Stow et al., 2009). For the sediment waves in Lake Brienz, we observe two different types: Type 1 is characterized by wave lengths of 100 to 200 m, with general wave heights of 1 to 3 m but may reach up to 8 m in the northwestern, distal area of the Aare Delta. Type 1 sediment waves are located in the southwestern distal area of the Aare Delta and northeastern distal part of the Lütschine Delta. These areas correspond to regions with a sand composition of 1–5% (Sturm, 1976). Type 2 is characterized by wave lengths of 25 to 75 m in the southern proximal area of the Aare Delta and 100 to 150 m in the northern proximal area of the Delta. Wave heights amount to 1 to 2 m, but can reach up to 6 m in front of the Aare Delta. At Lütschine, Type 2 wave lengths are 25 to 50 m close to the inflow and increase up to 150 m in more distal areas. Similarly, wave heights are around 1 m and reach up to 6 m in the northeast of the delta. The area of type 2 sediment waves typically shows 5–10% sand (Sturm, 1976). Within the active channels of both deltas, the wave lengths and heights are smaller and reach 10 to 25 m and 0.5 m, respectively. Typically, wave lengths tend to shorten towards the river inflows where flow velocities are higher.

**Fig. 3:**
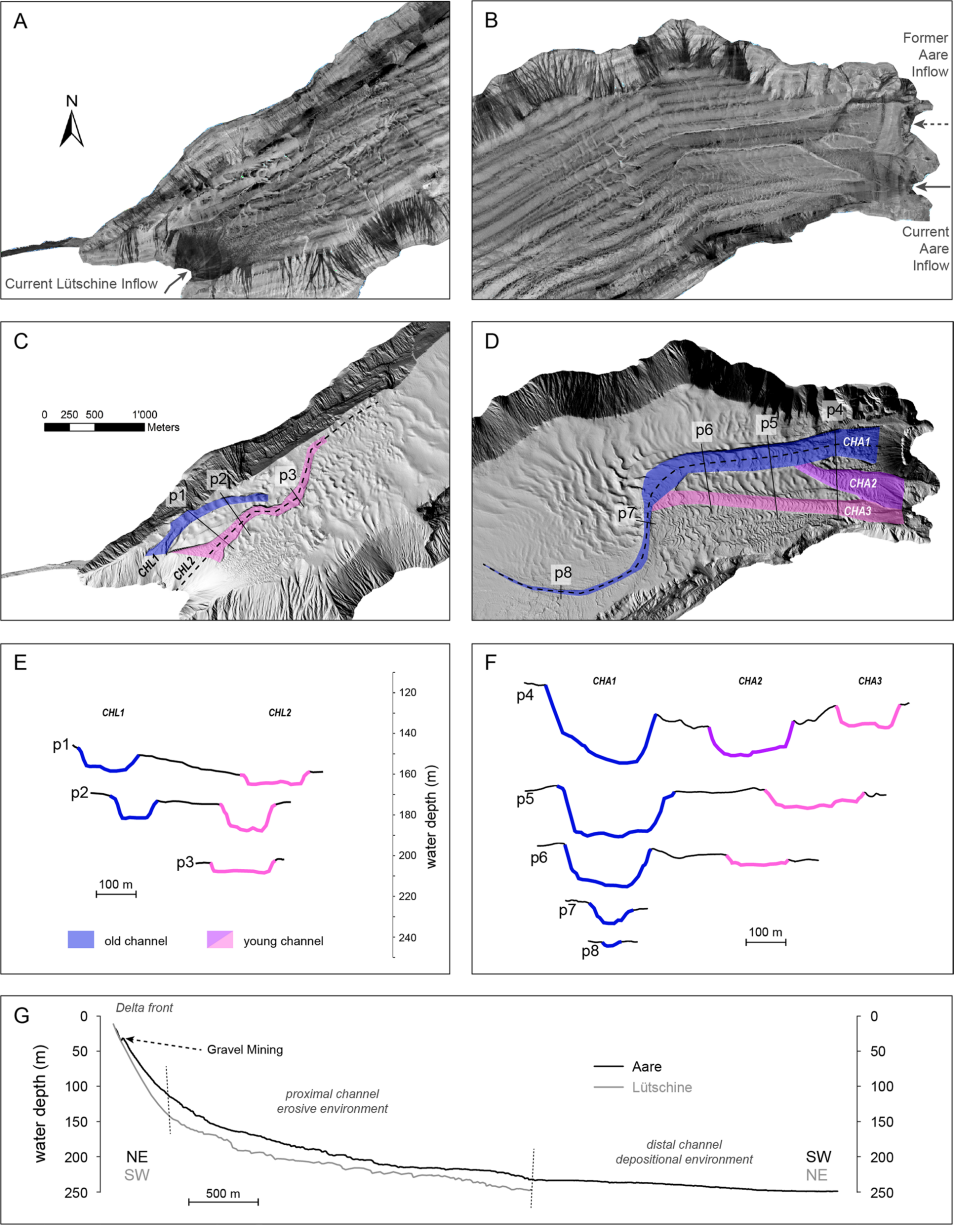
Subaquatic channel structures of Lütschine (**A**, **C**, **E**) and Aare Delta (**B**, **D**, **F**). A, B: Zoomed section of backscatter-intensity map. **C**, **D**: Hillshade with colours indicating different branches of the channels and solid black lines showing profiles (p1–p8) across the channels. **E**, **F**: Cross-sections of channels (p1–p8) at various depths (5-times vertically exaggerated). **G** Profiles along channel thalweg (5-times vertically exaggerated) marked as dashed lines in **C**-**D**

Backscatter intensity provides information on the substrate of the lake floor (Dartnell & Gardner, 2004; Hilbe et al., 2011), namely its acoustic hardness and roughness (Fig. 2B) and is commonly used for sea floor sediment facies analysis. In Lake Brienz, the subaquatic channels and the slopes yield highest backscatter intensities (− 20 to − 10 dB) and are clearly distinguishable from the basin plain (− 32 to − 20 dB). In the case of the steep slopes, we can expect the substrate to be hard rock and very irregular, yielding a strong backscatter signal. High-backscatter intensities in the channels can be attributed to coarse-grained material deposited on the channel floor. The basin plain echoes only a weak signal, which can be attributed to the very fine-grained muddy hemipelagic sediments that dominate in this area of the lake (Sturm und Matter, 1978). Towards the delta areas, the signal strength increases (Figs. 2B, 3A, B), in particular towards the Lütschine Delta, indicating dominance of fluvial-derived sandy deposits. Overall, the lake floor sediment facies interpretation based on bathymetric backscatter data is in good agreement with the sediment facies interpretation of Sturm (1976), who assigned sand proportions calculated from surficial sediment samples. The proximal delta areas, including the active channels are dominated by 5 to 50% sand matching intensity values of −20 to − 10 dB.

#### Subaquatic-channel system

Lake Brienz has two major subaquatic channel systems, which can be subdivided into different branches. Since both, the Aare and the Lütschine inflows were artificially redirected in the past, some of the channel branches are abandoned and are now inactive, hereafter termed “old channels”. After the river diversions, new active channels formed in both delta areas (Fig. 3C, D).

In the Aare Delta, the young channel, at the present inflow of the Aare, divides into a northern (CHA2) and southern (CHA3) branch, both merging into the old channel (CHA1) further downstream (Fig. 3D). Hence, CHA1 remains partially active and only the uppermost part close to the former river inflow turned inactive. The abandoned part of the CHA1 extends over a length of 4.8 km and is deeper incised than its younger counterparts (Fig. 3F). Following the downstream evolution of the delta, three sections can be distinguished: delta front, proximal channel area and distal channel area (Fig. 3G). On the delta front, typical foreset bedding on steep slopes with ~ 14° inclination occur. Notable is a small step in this part of the profile, which can be attributed to gravel mining at the former river mouth of the Aare. In the proximal channel area, the erosive power is highest with channels incising more than 20 m into the substrate (p4–7; Fig. 3F). Here, average slope inclination is 2.7°. Characteristic features are the upward-bent dune morphologies on the channel floor and the steep channel walls with explicit levee structure on either side of the channel. The proximal channel area is also clearly visible on the backscatter image (Fig. 3B) indicating gravel to sandy deposits in this part of the channel. The northern channel walls (p4-p7; Fig. 3F) are always more pronounced and larger in overall height than its southern analogue. This most likely can be attributed to the Coriolis force deviating the current to the right when flowing downstream, leading to enhanced erosion in the northern part of the channel. This is in agreement with observations in other subaquatic channel-systems such as in Lake Geneva (Sastre et al., 2010). With increasing distance from the river inflow, the channel narrows and the channel wall height decreases. There are also overspill structures occurring before the bend of the channel (Fig. 2C). Thereafter, the channel starts incising again before it gradually levels off towards the lake basin. In this distal part of the channel slope, inclination does not exceed 0.7°.

In the Lütschine Delta, channels are smaller than the Aare channels, reaching a maximum length of 1.8 km (Fig. 3C). The young channel (CHL2) does not merge with the old channel (CHL1) and rather cuts the old channel, accompanied by pronounced incision with a max. depth of up to 12 m (Fig. 3E; p2). With an average slope angle of 18.4°, slope inclinations in the delta front area are steeper for the Lütschine Delta (Fig. 3G) than for the Aare Delta suggesting higher abundance of coarse-grained deposits. The backscatter image confirms this conclusion (Fig. 3A) with highest intensities at the front of the Lütschine Delta. This is also in agreement with the suggestion of Sturm (1976), reporting more than 50% sand content in the most proximal area of the Lütschine Delta.

### Seismic data

#### General seismic stratigraphy

**Fig. 4:**
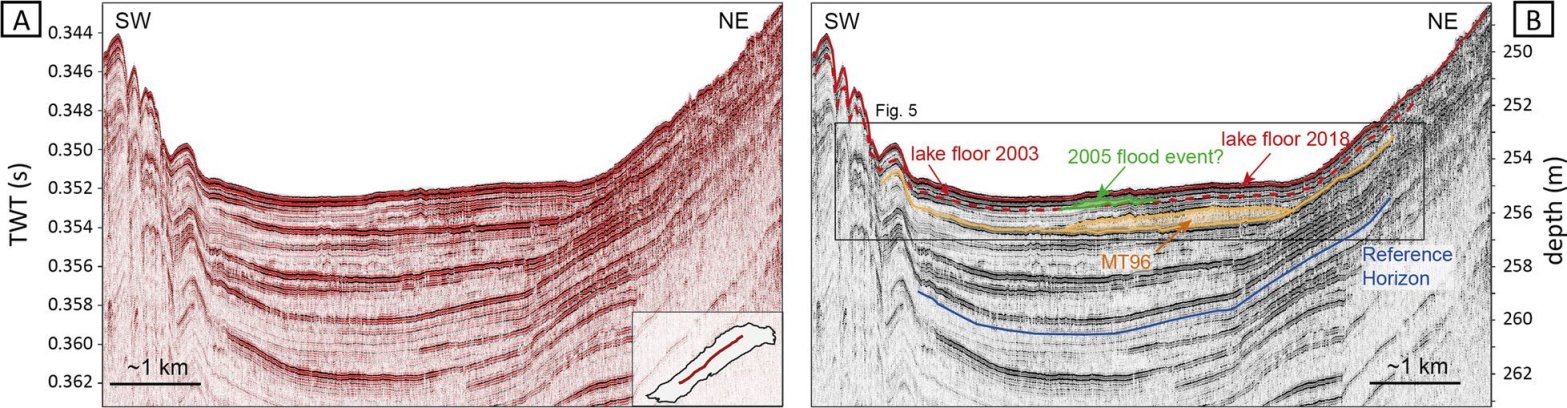
Longitudinal transect of the Lake Basin as obtained from seismic data in 2018 (see inset for exact location of the reflection seismic profile). Uninterpreted seismic profile is depicted on the left. Key features like megaturbidite deposits (MT96) and current lake floor are highlighted on the right. Reference horizon for bulk shifting (synchronization between vintages) is marked in blue

**Fig. 5:**
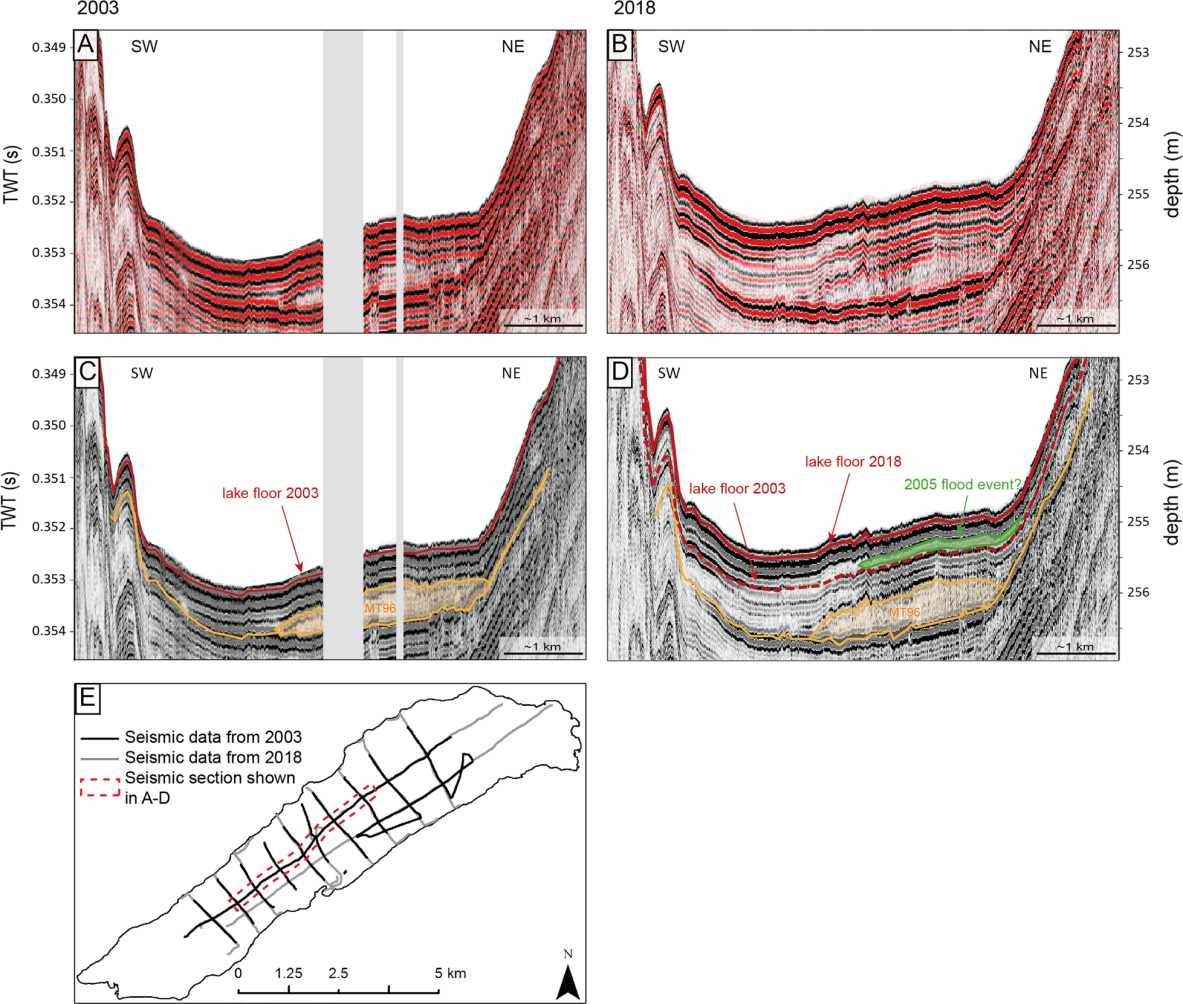
Quasi-4D-Seismic reflection profile (see Fig. 4 for location) from this study (**B**, **D**) being compared with reflection seismic profiles from Girardclos et al. (2007) (**A**, **C**). Uninterpreted seismic data are depicted on the top. Grey bars represent sections where no data is available

A reflection seismic profile on a longitudinal transect of the lake (Fig. 4) shows good signal penetration of up to 55 ms TWT or ~ 40 m. Closer to the delta areas, seismic penetration is prevented by free gas in the deposits of the major tributaries. The seismic stratigraphy is dominated by two seismic facies (i) a few medium-amplitude, parallel and continuous reflections, interpreted as hemipelagic background sedimentation with few thinner event deposits with thicknesses below the threshold of seismic resolution (~ 10 cm), and (ii) semi-transparent to chaotic seismic reflections with a high-amplitude reflection at their base that can be interpreted as megaturbidite deposits (MT96; e.g. Schnellmann et al., 2006; Girardclos et al., 2007; Kremer, Hilbe, et al., 2015). These deposits exceed 10 cm in thickness and can reach a maximum thickness of 2 m. The uppermost megaturbidite (MT96) can be linked to a spontaneous collapse of the Aare Delta in 1996 (Girardclos et al., 2007). Furthermore, faintly visible reflection downlapping towards the basin plain and topped by a semi-transparent reflection, can be observed at the base of the post-2003 seismic data (Figs. 5B, D). Although not clearly visible, as these reflections are at the limits of the seismic resolution, we interpret these as river-derived flood deposits representing a major flood event.

#### Sedimentation rates from quasi-4D seismic data

**Fig. 6:**
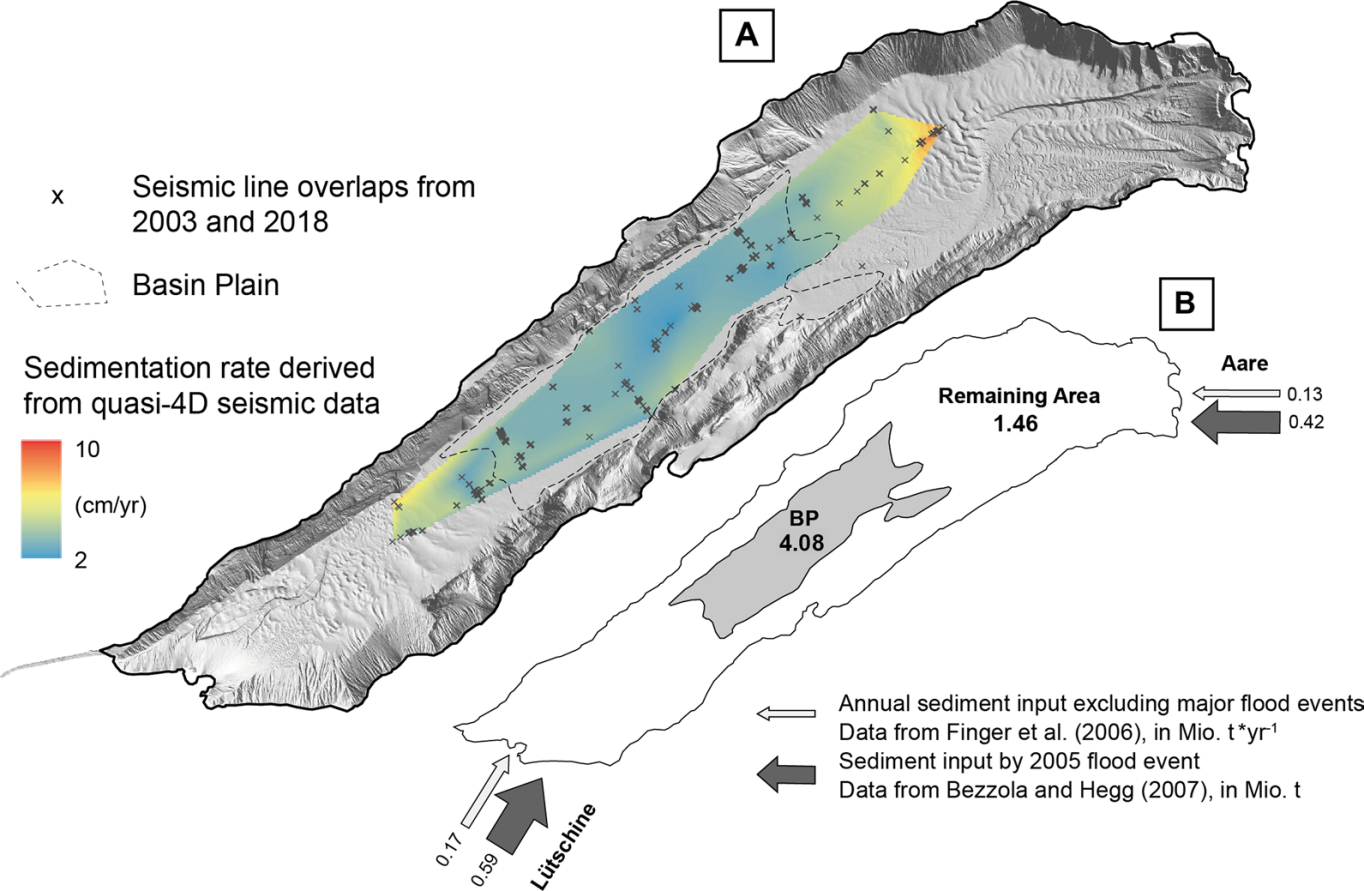
**A** Sedimentation rates as interpolated from seismic data from 2003 (Girardclos et al., 2007) and 2018 (this study). Crosses indicate overlapping of the reflection seismic profiles where sedimentation rate could be calculated directly. **B** The simplified sketch shows the sediment dynamics in Lake Brienz. Numbers represent sediment loads [Mio t] as calculated for the time period 2003–2018. Arrows indicate mean annual sediment input [Mio t/yr] and additional sediment input [Mio t] corresponding to the 2005 flood event of the main tributaries

A comparison with seismic data from 2003 (Girardclos et al., 2007) allows detailed quantification of sedimentation over the past 15 years (Fig. 6A). It becomes evident that the lake floor 2018 (352.6 ms at a water depth of 255.6 m) is significantly shallower compared to 2003 (353.3 ms at a water depth of 256.1 m; Fig. 5). Throughout the lake basin, average sediment accumulation amounts to 43.5 cm over a period of 15 years, corresponding to an average sedimentation rate of 3.0 cm/yr (Table 3). Lowest sediment accumulation occurred in the central basin with sediment thickness of ~ 30 cm (2.0 cm/yr). Towards both delta areas, the thickness of the post-2003 sediments increases to a maximum of 70.5 cm indicating a sedimentation rate of up to 4.7 cm/yr. Total sediment volume considering only the area of the basin plain amounts to 2.2 × 10^6^ m^3^ (Table 3) corresponding to a sediment dry mass of 4.08 × 10^6^ t. The average sediment density was derived from Girardclos et al. (2007) using a grain density of 2.65 g/cm^3^ and an average porosity of 70% (graded silt) resulting in an average density of 1.86 g/cm^3^, which is applied to the sediment volume of the basin plain to obtain the sediment dry mass. The calculated sedimentation rates and volumes, however, do not only enclose hemipelagic sedimentation, but also mass-movement deposits and flood events such as for example the 2005 flood event that caused an important sediment supply into Lake Brienz as shown by Bezzola and Hegg (2007; see also Sect. 4.3). The delta-derived downlapping and basinward-thinning sediment wedge as described above (in 4.2.1) is interpreted to relate to this event.

**Table 3:** Accumulated sediment in the basin plain within the last 15 years derived from seismic (A) and gauging data (B)

A) Seismically derived	
Mean sedimentation rate	0.03 m/yr
Total basin plain area	5.05 × 10^6^ m^2^
Mean sediment accumulation within 15 yrs	15 yr × 0.03 m/yr = 0.435 m
Sediment volume	0.435 m × 5.05 × 10^6^ m^2^ = 2.2 × Mio m^3^
Sediment dry mass in 15 years (incl. event deposits)	1855 kg/m^3^ × 2.2 Mio m^3^ = 4.08 Mio t
B) Instrumentally derived (suspended load)	
Avg. river input: within 15 years: 2005 flood input: (Bezzola & Hegg, 2007)	0.302 Mio. t yr^−1^ 15 yr × 0.302 Mio. t yr^−1^ = 4.53 Mio. t 0.42 + 0.59 Mio. t = 1.01 Mio. t
Total river input:	4.53 + 1.01 Mio. t = 5.54 Mio. t

### River-derived sediment budgets in the basin plain

Sediment yield can be compared directly to riverine sediment input of the main tributaries from instrumental dataset, as other sources of sediment input are neglectable (Sturm, 1976). Mean annual sediment load of the rivers as calculated for an 8-year time period without major flood events (1997–2004; Finger et al., 2006) amount to a total sediment input of 0.302 × 10^6^ t/yr (Aare: 0.13 × 10^6^ t/yr; Lütschine: 0.17 × 10^6^ t/yr, Fig. 6B). For a 15-year time period, as covered in this study (2003–2018), normal background sedimentation (Finger et al., 2006) yields 4.53 × 10^6^ t of sediment. This time period, yet includes several minor flood events, however the 2005 flood event, is considered as the one that brought highest amounts of sediment to the lake. Bezzola and Hegg (2007) reconstructed sediment volumes of the main tributaries during the 2005 event to be 3–4 orders of magnitude higher as compared to annual loads and amounting to a total sediment input of 1.01 × 10^6^ t (Aare: 0.42 × 10^6^ t; Lütschine: 0.59 × 10^6^ t). Thus, total river-derived sediment input for the time period 2003–2018 amounts to 5.54 × 10^6^ t including the 2005 flood event. If we compare the total river-derived sediment input (5.54 × 10^6^ t) with the sediment amount deposited on the basin plain (4.08 × 10^6^ t, see Sect. 4.3), ~ 75% of the river input is deposited on the basin plain and ~ 25% is deposited on the remaining area (delta area and steep basin slopes).

## Discussion

### Sedimentation pattern in the delta area

Most freshwater systems lack well-developed channel-systems as observable in Lake Brienz (Talling, 2014). Many perialpine lakes such as Lake Zurich are fed by small rivers that are unable to form major delta areas (Strupler et al., 2018). Apart from water discharge, also sediment load of the tributaries plays a crucial role for the formation of subaquatic channels (Mulder et al., 2003; Talling, 2014). As demonstrated by several studies, subaquatic channels are formed by high-density currents also termed underflows or turbidity currents, which are capable of eroding the underlying substrate (e.g. Corella et al., 2014; Lambert & Giovanoli, 1988; Mulder et al., 2003; Talling, 2014). The most erosive and powerful underflow events are triggered by slope failures (Lambert & Giovanoli, 1988; Girardclos et al., 2007; Fanetti et al., 2008).

Similar subaquatic channel systems as described here in Lake Brienz can be observed in few other (peri-)alpine lakes (Girardclos et al., 2012; Wessels et al., 2010). In Lake Geneva, nine channels have been observed in the Rhone Delta, resulting from natural or anthropogenic shifting of the river mouth (Loizeau et al., 2012; Sastre et al., 2010). The most prominent channel can be attributed to the current Rhone inflow. With a length of 13.5 km and a maximum depth of ~ 50 m (Sastre et al., 2010), it is more than twice the size of CHA1 in Lake Brienz. On a similar range of order as in Lake Geneva are the channels of the old Rhine Delta in Lake Constance extending over a distance of 15.5 km and incising up to 70 m into the substrate (Wessels et al., 2010). Such larger channel systems show features that are not present in the delta areas of Lake Brienz like explicit meandering structures of the channel or terminal lobe structures. Similar structures, however, comprise the steep delta front, current and overspill structures, crescentic bedforms on the channel floor as well as older abandoned channels from previous river inlets (Corella et al., 2014; Wessels et al., 2010). In the following, several depositional and erosive processes and their resulting morphologies will be discussed in detail.

The highest sediment accumulation rates are observed at the delta front: From comparison of two bathymetric maps of Lake Geneva, Silva et al. (2019), demonstrated that this area of the lake yields average sedimentation rates of 7.37 cm yr^−1^. The planar shape of this part of the delta area is attributed to the coarse-grained river load deposit resting at the angle-of-repose. Slope inclination depends on sediment texture and grain size of the riverine sediments (Adams et al., 2001). The delta front can also be the source of large mass-movement events (Kremer, Hilbe, et al., 2015; Talling, 2014) such as the 1996 megaturbidite deposit that has been traced back to a slope failure of the Aare Delta (Girardclos et al., 2007). However, in our study, we could not detect any geomorphologic traces like failure scars as remnants of such a massive event. We assume that due to high sedimentation rates and rapid delta progradation of the Aare Delta, traces of this event have been refilled. Other morphologies of net-accumulation are levees and the distal lobe complex (Silva et al., 2019). No terminal lobe occurs in Lake Brienz and levees are less explicit as observed in other subaquatic channel complexes (e.g. Corella et al., 2016) and probably play a minor role in sediment budgeting of the delta areas. Overspill structures along the channel bend in CHA1 suggest that turbidity currents frequently leave the confined channel walls and deposit most of their sediment in the sinuous shaped sand waves (see Hay, 1987; Hiscott et al., 1997 for more details on this mechanism).

Net-erosion occurs within the subaquatic channels (Silva et al., 2019). A four-months daily bathymetric survey of the Squamish Delta, a fjord-type like delta with submarine channel in British Colombia, showed extreme incompleteness of stratigraphic record of subaquatic channels: median preservation is not more than 11% due to underflows constantly reworking the sediment (Vendettuoli et al., 2019). Characteristic morphologic features signalizing strong currents within the channels are the upward bent dune morphologies, which have been described by several authors (e.g. Hughes Clarke, 2016; Normandeau et al., 2019; Slootman & Cartigny, 2020; Symons et al., 2016). Those morphologies are interpreted as upstream-migrating bedforms indicative of supercritical turbidite flows that only occur in confined channel systems (Stow et al., 2009; Symons et al., 2016 and references therein). Direct measurements of such turbidites have been performed by Lambert and Giovanoli (1988) in Lake Geneva during a 78-day measurement campaign. The five largest events reached flow velocities of > 0.5 m s^−1^. It can be expected that bigger events like delta-slope failures reach even greater speeds.

Nevertheless, human intervention in the catchment area of a lake such as hydropower damming, river deviation and channelizing, sediment mining or land-use change might have several impact on the sediment regime in delta areas (e.g. Girardclos et al., 2007; Lane et al., 2019; Vörösmarty et al., 2003; Wirth et al., 2011). Alpine lakes such as Lake Brienz belonging to the first major sedimentary sinks, usually react more directly to disturbance than lowland and marine settings. The latter comprise larger catchments and react with significant time lags in their response (Dearing & Jones, 2003; Thevenon et al., 2013). In Lake Brienz, sedimentation in the Aare Delta is mainly altered by human intervention. Hydropower damming significantly reduced the coarse-grained deltaic sedimentation (Anselmetti, Bühler, et al., 2007). Moreover, water retention in the reservoir reduces frequencies of flood event with elevated particle concentration (Finger et al., 2006). Consequently, the density of the Aare water is lowered and plunging events are less frequent. The same effect occurs in Lake Geneva (Loizeau & Dominik, 2000): the erosive power of underflows significantly diminished following the construction of hydroelectric reservoir in the upstream Rhone River catchment. From 1994 to 2009, the within-river sediment mining significantly reduced sediment concentration and loads to Lake Geneva and hence likely further increased this tendency (Lane et al., 2019). The Lütschine Delta in contrast remains unaffected of hydropower damming (Anselmetti, Bühler, et al., 2007). This might explain the coarser grain size in the delta front as indicated by high backscatter intensities and steeper slope inclination (Fig. 3A, B, G). In addition, human interventions such as deforestation (Arnaud et al., 2005; Mueller & Loew, 2009) or drainage of wetlands for gain of cultivated land (Schulte et al., 2009) that induce soil erosion can lead to significant pulse of sediment supply into lakes. Higher denudation rates in the catchment lead to higher terrigenous fluxes to the lake (Thevenon et al., 2013). Carvalho and Schulte (2013) pointed out that increased soil erosion due to land-use change combined with an effective river management (embankment and channelization) in the lower catchment of the Aare resulted in a more efficient transport of sediment to the lake.

### Sedimentation pattern in the basin plain

The datasets of this study show that 17% of the lake floor (basin plain) accommodates ~ 75% of the total riverine sediment input when comparing seismic reflection and instrumental data. An average sedimentation rate of 3.0 cm/yr as calculated above for the basin plain in Lake Brienz is exceptionally high if compared to rates from previous studies from the same basin. For example, Anselmetti, et al. (2007) calculated significantly lower sedimentation rates of 1.1–1.6 cm/yr for the basin plain of Lake Brienz covering a time span without major flood events (1996–2003). Therefore, the difference between the sedimentation rate estimated by Anselmetti, Bühler, et al. (2007) and our can be partly explained by the excess sediment input of flood events such as the 2005 flood event, known from instrumental datasets and imaged in the reflection seismic data (Fig. 5D). This centennial flood has deposited 1.01 × 10^6^ t of sediments as estimated by instrumental datasets. This represents ~ 1/5 of the total riverine sediment input over 15 years, and i.e. thus is equivalent to ca. 3 years of the mean input for this period.

Nevertheless, also differences in methodology may account for the significantly higher sedimentation rates as calculated for this study. Sedimentation rates are often derived directly from sediment cores allowing, in combination with age-dating methods, precise calculations. However, mechanical impact of the coring device can lead to compaction and loss of the uppermost sediments (Crusius & Anderson, 1991; Loizeau et al., 2012). Furthermore, degassing processes in cores might lead to an overestimation of sedimentation rates. Non-invasive techniques such as seismic investigations, on the other hand, do not disturb the sediment. However, they have significantly lower resolution with an average error of ± 5 cm for the uppermost sediments. Furthermore, small mass movements in the range of few centimetres thickness cannot be detected as they are beyond the resolution threshold of the seismic source. In this case, normal background sedimentation rates would be overestimated. Nonetheless, there are many advantages using seismic measurements. Whereas cores represent only point measurements, quasi 4D-seismic quantifications provide approximations on a basin-wide scale and thus, allows sediment budgeting as done for this study.

Nevertheless, sedimentation rates of more than 1 cm/yr in the basin plain are exceptionally high as becomes evident when compared to other (peri-)alpine lakes (Table 4) and usually can only be found in delta areas (e.g. Schröder, 1992; Silva et al., 2019). Mostly sedimentation rates in the basin plain exhibits values in the range of few millimetres per year as has been demonstrated e.g. for Lake Zurich or Lake Lucerne (Hilbe et al., 2011; Lister et al., 1983; Strasser et al., 2013). Similar values were also reported for Lake Como, Lake Iseo as well as for distal parts of Lake Geneva or Lake Constance (Bini et al., 2007; Fanetti et al., 2008; Loizeau et al., 2012; Schröder, 1992). Also, further downstream, Lake Thun and Lake Biel, both connected with Lake Brienz by the Aare River, yield lower basinal sedimentation rates ranging between 0.4 and 0.9 cm/yr. Similar sedimentation rates as measured in Lake Brienz, on the other hand, could be demonstrated e.g. for the southern basin of Lake Traunsee (Schneider et al., 1986). This part of the lake resembles closely Lake Brienz’ morphology with steep lateral slopes and a major delta area on one end of the basin. Furthermore, river Traun, similar to Lütschine and Aare, brings high amount of allochthonous sediment to the lake. Also, Lake Uri (southern basin of Lake Lucerne) exhibits high sedimentation rates. However, sedimentation rates as calculated by Siegenthaler and Sturm (1991) cannot directly be compared to those of Lake Brienz since they take sediment porosity into account.

**Table 4:** Compilation of sedimentation rates and sediment accumulation rates in various (peri-)alpine lakes. Sedimentation rates were calculated based on different methods like cores combined with age dating, seismic data and/or bathymetric data

Lake	Sedimentation rate (cm yr^−1^)	Location within the lake	References
Lake Biel	0.86	Southern Basin from 52 water depth	Thevenon et al., (2013)
Lake Brienz	3.0 1.06–1.61 0.71–1.18 1.17–2.29 0.26–0.68	Basin plain Basin plain Aare Delta Lütschine Delta Iseltwald Basin	This study Anselmetti, Bühler, et al. (2007), Anselmetti, Hodell, et al. (2007)
Lake Como	0.42–0.73	Deep Basin	Fanetti et al., (2008)
Lake Constance	0.4 0.1–0.4 0.38	Obersee Obersee (NS-transect) Central Basin Obersee	Schröder, (1992) Wagner et al., (1998) Fabbri et al., (2021)
Lake Geneva	7.37 2.59 2.46 0.90	Delta front Canyon and levees Distal lobe Transition zone to Lake Basin	Silva et al., (2019)
Lake Iseo	0.1	Average value from seismic data	Bini et al., (2007)
Lake Lucerne	0.1 0.02–0.05	Basinal area lacking major delta areas Gently dipping lateral slopes	Schnellmann et al., (2006) Strasser et al., (2007) Hilbe et al., (2011)
Lake Thun	0.4–0.78 0.17–0.48 0.4–0.6 0.6–0.9	Near Kander Delta On the shore terrace Central plain Lateral slopes	Sturm and Matter, (1972) Wirth et al., (2011)
Lake Traunsee	2–3 0.4	Southern basin Northern basin	Schneider et al., (1986)
Lake Zurich	0.04	Basin plain	Lister et al., (1983)

Three reasons can account for extraordinarily high sediment yield in the basin plain of Lake Brienz:

1. *First major sedimentary sink*
  Thevenon et al. (2012) demonstrated that Lake Brienz, being the first major sedimentary sink, receives up to three times more sediment input than further downstream Lake Thun or Lake Biel, all connected by the Aare River. Half of the lake’s catchment is situated above 2000 m a.s.l. with 19% of its area being glaciated bringing high amounts of glacial meltwater and clastic sediment to the lake (Jordi et al., 2006; Wüest et al., 2007).
2. *High connectivity between delta areas and basin plain*
  Due to its small, elongated shape with two productive delta areas on either side of the lake, the morphology of the lake allows an efficient transport of sediment to the lake basin. Larger lakes, such as e.g. Lake Geneva or Lake Constance, have a much more expanded flat basin contributing to most of the lake’s surface. Thus, only major mass-movement events reach the far distal parts of the basin plain (Kremer, Hilbe, et al., 2015)
3. *Subaquatic channel-system*

As has been shown by several studies (e.g. Corella et al., 2014; Silva et al., 2019; Vendettuoli et al., 2019), subaquatic channels as present in Lake Brienz act as effective conduits transferring sediment to the basin plain. Moreover, they act as important sediment source themselves with underflow events constantly reworking sediment of channel floor and walls (Silva et al., 2019; Vendettuoli et al., 2019). Lakes lacking channel systems, on the other hand, deposit all there sediment in prodeltas and delta fronts (e.g. Fanetti et al., 2008).

## Summary and conclusions

By the combination of newly acquired high-resolution bathymetric data and reflection seismic profiles, new insights into the geomorphology of Alpine Lake Brienz and its sediment dynamics could be revealed. Multibeam bathymetric data suggests three distinct geomorphologic areas: (i) slopes with intercalated terraces, (ii) flat basin plain and (iii) delta areas of Lütschine and Aare Rivers with subaquatic channels. A quasi-4D comparison of seismic data from this study with older vintages from 2003 allows budgeting of sediment accumulation in the past 15 years (2003–2018) indicating that:

- Total sediment input into the lake amounts to 5.54 × 10^6^ t of which ~ 75% are deposited in the basin plain (~ 17% of the total lake-floor area) directly
- Extraordinarily high average basinal sedimentation rates of up to 3.0 cm/yr are observed, as compared to other (peri-)alpine lakes
- The 2005 flood event is responsible for ~ 1/5 of the total sediment input over 15 years
- Lake Brienz yields one of the highest sediment supplies to the basin plain due to its high-alpine catchment, and subaquatic channel systems funneling sediment transfer to more distal parts of the lake.

Three possible reasons account for the extraordinarily high sediment yield in Lake Brienz: i) the proximity to its high-alpine catchment area, ii) the combination of a high connectivity between the delta and the basin plain, and iii) the efficiency of conduits transferring sediment to the basin plain (subaquatic channel-system). We further note that the deltaic sedimentation is mostly controlled by its subaquatic channels but also highly altered by human intervention.

## Data Availability

The datasets generated during and/or analyzed during the current study are available from the corresponding author on reasonable request.
